# Percutaneous kyphoplasty with or without posterior pedicle screw fixation for the management of severe osteoporotic vertebral compression fractures with nonunion

**DOI:** 10.1186/s13018-024-04714-y

**Published:** 2024-04-15

**Authors:** Yingchuang Tang, Hanwen Li, Xingbang Ruan, Huilin Yang, Jiajia Sun, Kangwu Chen

**Affiliations:** First Affiliated Hospital of Soochow University, Suzhou, China

**Keywords:** Osteoporosis, Percutaneous kyphoplasty, Severe osteoporotic vertebral compression fracture, Fracture nonunion, Posterior pedicle screw fixation

## Abstract

**Objective:**

To assess the radiographic outcomes, clinical outcomes and complications of percutaneous kyphoplasty (PKP) with and without posterior pedicle screw fixation (PPSF) in the treatment of severe osteoporotic vertebral compression fractures (sOVCF) with nonunion.

**Methods:**

This study involved 51 patients with sOVCF with nonunion who underwent PKP or PPSF + KP. The operation time, intraoperative blood loss, volume of injected bone cement, operation costs and hospital stays were all recorded. In addition, the Visual Analogue Scale (VAS) and the Oswestry Disability Index (ODI) were assessed separately for each patient before and after surgery.

**Results:**

Compared with the PPSF + KP group, the PKP group had shorter operation time, less intraoperative blood loss, shorter hospital stays and fewer operation costs. However, cobb’s angle improvement (13.4 ± 4.3° vs. 21.4 ± 5.3°), VWR improvement ratio (30.4 ± 11.5% vs. 52.8 ± 12.7%), HA (34.9 ± 9.0% vs. 63.7 ± 7.6%) and HM (28.4 ± 11.2% vs. 49.6 ± 7.7%) improvement ratio were all higher in PPSF + KP group than that in PKP group. In addition, the ODI index and VAS score in both groups were significantly decreased at the postoperative and final follow-up. PKP group's postoperative VAS score was significantly lower than that in PPSF + KP group, but there was no statistically significant difference in VAS score at the last follow-up.

**Conclusion:**

PKP and PPSF + KP can both effectively relieve the pain associated with sOVCF with nonunion. PPSF + KP can achieve more satisfactory vertebral reduction effects compared to PKP. However, PKP was less invasive and it has more advantages in shortening operation time and hospital stay, as well as decreasing intraoperative blood loss and operation costs.

## Introduction

One of the most common complications of osteoporosis is osteoporotic vertebral compression fractures (OVCF) [1, 2]. It can cause severe back pain, limit daily activities, and increase the risk of systemic complications [3, 4]. As minimally invasive spinal surgery procedures improve, an increasing number of patients with osteoporotic vertebral compress fractures who have failed conservative treatment will be willing to undergo surgery. During these procedures, lots of severe OVCF (sOVCF) with nonunion were observed. Nonunion does not heal over time, and pain symptoms may gradually worsen, negatively impacting quality of life [5]. Traditional surgery aimed to correct kyphosis, achieve fixation and fuse the vertebrae, but it resulted in more paravertebral muscle and ligament destruction, as well as increased intraoperative blood loss [6]. Worse, internal fixation has a high failure rate in patients with severe osteoporosis [7]. Severe osteoporotic vertebral compression fractures with nonunion were once considered a relative contraindication to PKP [8, 9]. Nowadays, sOVCF with nonunion are no longer contraindicated for PKP due to advancements in surgical techniques and higher-definition imaging systems [10]. We analyzed and compared the safety and efficacy of PKP and PPSF + KP in the management of sOVCF with nonunion in our study.

## Materials and methods

This was a single-center, retrospective study. Our institute recruited sOVCF patients with nonunion who underwent PKP or PPSF + KP between February 2017 and September 2020. The demographic information for the two groups is shown in Table 1. After evaluation by the team of surgeons, all patients had good lumbar stability and the two surgical options were chosen by the patients. Traditionally, patients were fully informed about the advantages and disadvantages of both surgical methods prior to surgery. The choice of surgery is guided by the surgeon, who chooses the most appropriate surgery for the patient after fully analyzing each patient.

**Table 1:** Demographic data of patients

Variable	PKP group	PPSF + KP group	P value
N	27	24	–
Gender (M/F)	7/20	6/18	0.940
Age (Y)	75.19 ± 5.94	75.33 ± 6.62	0.933
Course of disease (Mo)	3.81 ± 2.29	3.50 ± 1.69	0.583
BMD	− 3.49 ± 0.49	− 3.63 ± 0.32	0.236
Responsible segment (N)			
T10	1	1	0.557
T11	0	1	
T12	8	7	
L1	10	9	
L2	5	4	
L3	2	2	
L4	1	0	
Follow-up (Mo)	27.04 ± 2.08	27.17 ± 2.07	0.825

Values are expressed in mean ± standard deviation

*N* number, *M/F* Male/Female, *Y* years, *Mo* months, *BMD* bone mineral density

### Inclusion and exclusion criteria

The inclusion criteria were as follows: (1) Patients who met the diagnostic criteria for sOVCF with nonunion had the height of the fractured vertebral body collapsed to one-third or less of its original height, meanwhile, based on clinical complaints and imaging evaluation, low T1 and high T2 signal on MRI, and fracture line widening on routine radiographs; (2) T value < 2.5 for bone density on dual-energy Xray absorptiometry, which is consistent with the symptoms; (3) Magnetic resonance imaging (MRI) and symptoms and signs revealed no signs of nerve injury; (4) Only one responsible vertebra is involved [11, 12].

The exclusion criteria were as follows: (1) Patients with pathological vertebral fractures, multiple vertebral fractures or severe internal medical diseases such as spinal metastatic tumors; (2) Patients who are unable to tolerate surgery due to severe cardiopulmonary, liver, or kidney dysfunction; (3) Patients with incomplete clinical information.

### Surgical technique

Under general anesthesia, patients in PKP group were carefully prepared in a prone position with a lordotic posture to keep the spine in a posterior extension. The injured vertebra was located and the surgical was monitored by C-arm fluoroscopy. Following bilateral transpedicular puncture under general anesthesia, the working tunnel was used to place balloons beneath the endplate. After elevating the endplate, balloons were gently inflated and deflated to help restore vertebral height. After removing balloons, the polymethyl methacrylate (PMMA) cement with toothpaste-like viscosity was slowly injected into the cavity until it was completely filled. Our hospital used incremental temperature cement delivery and graded infusion techniques to reduce leakage [11, 13].

Patients in PPSF + KP group were sedated and placed in a prone position. Preoperatively, to restore spine hyperextension, pillows were placed under the diseased vertebra. We used a standard posterior midline approach to expose spinous process, lamina, and subtalar joints before inserting four pedicle screws into the adjacent superior and inferior vertebrae of the operated vertebrae to restore the height of the fractured vertebrae via positioning, internal fixation bracing, and lateral elevation [14]. Following that, 2 rods were used to secure 4 pedicle screws. Following a similar procedure to PKP, balloons were used to thoroughly expand the compressed vertebral body before the PMMA cement was progressively injected. The purpose of PPSF was to effectively stabilize the fractured vertebrae and provide support for healing of the fractured vertebrae, not to fuse the vertebrae.

### Clinical evaluation

All clinical evaluations were performed before and after surgery and postoperative follow-up data were analyzed for both groups. X-rays were routinely performed prior to surgery and at all postoperative follow-ups, and CT and MRI images were analyzed as needed. The operation time, intraoperative blood loss, hospital stays, volume of injected bone cement, operation costs, the Oswestry Disability Index (ODI), the Visual Analogue Scale (VAS), the vertebral height (FVBH), the cobb's angle, and the VWR improvement ratio, as well as surgical complications, were all recorded and analyzed for the two procedures. All radiological measurements were double-blindly performed by two orthopedic surgeons.

### Statistical analysis

SPSS (Statistical Package for Social Sciences) version 21.0 (IBM SPSS, Chicago, IL, USA) statistical software was used for the study. The Student's t-test is used to compare continuous variables that are reported as mean ± SD. Pearson's chi-squared test is used to compare categorical data given as a number (%). The significance level was chosen at P < 0.01 or P < 0.05.

## Results

All 51 patients underwent successful surgery and were followed up for at least 24 months (range 24–43 months). There were no significant differences in the duration of symptoms or the follow-up period between the two groups. In addition, the demographic characteristics between the two groups was not significantly different (Table 1).

All patients tolerated the operation well. In both groups, there was a significant improvement in back pain after surgery. Table 2 depicts the intraoperative conditions of the two groups. Compared with PPSF + KP group, PKP group had significantly shorter operation time (41.81 ± 4.04 min vs. 116.71 ± 15.46 min, p < 0.01), less intraoperative blood loss (10.19 ± 1.80 ml vs. 90.21 ± 15.36 ml, p < 0.01), less operation costs (43,256.3 ± 5351.8 RMB vs. 89,607.3 ± 17,210.6 RMB, p < 0.01), and shorter hospital stays (3.52 ± 0.51 vs. 8.92 ± 1.69, p < 0.01). In PKP and PPSF + KP groups, the average cement injection volume was 6.04 ± 0.85 mL and 6.96 ± 0.81 mL, respectively.

**Table 2:** Comparison of intraoperative outcomes between the two groups

Variable	PKP group	PPSF + KP group	P value
Operative time (Min)	41.81 ± 4.04	116.71 ± 15.46	< 0.01
Intraoperative blood loss	10.19 ± 1.80	90.21 ± 15.36	< 0.01
Operation costs	43,256.30 ± 5381.83	89,607.61 ± 17,210.64	< 0.01
Cement volume (mL)	6.04 ± 0.84	6.96 ± 0.81	< 0.01
Hospital stays (d)	3.52 ± 0.51	8.92 ± 1.69	< 0.01

**Fig. 1:**
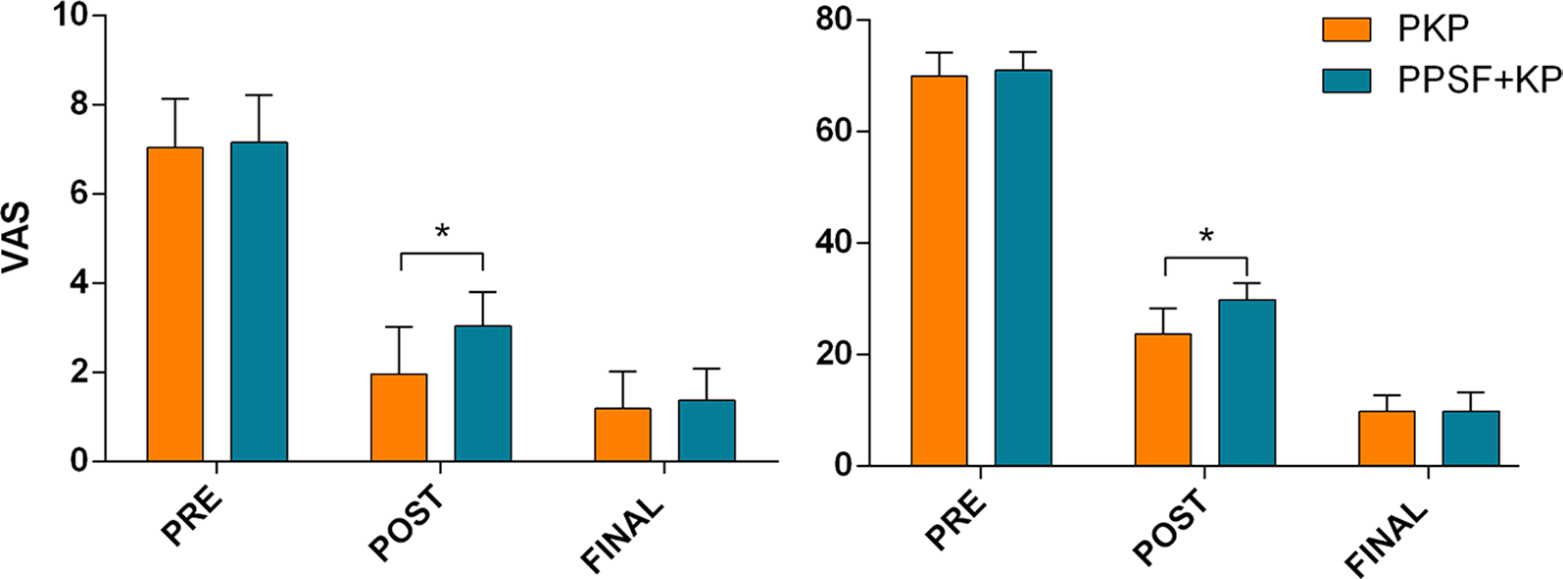
Comparison of the Visual Analogue Scale (VAS) and the Oswestry Disability Index (ODI) scores between the two groups. *Significance between the two groups, P < 0.05. Values are expressed in mean ± standard deviation

Patients' functional improvement was satisfactory, and symptoms improved at various time points after surgery in both groups. When compared to preoperative values, there were significant improvements in both groups' VAS and ODI scores at postoperative and final follow-up (p < 0.01) (Fig. 1). In PPSF + KP group, the mean VAS scores decreased from 7.17 ± 1.05 at preoperative to 3.04 ± 0.75 at postoperative, finally 1.38 ± 0.711. In PKP group, the mean VAS scores decreased from 7.04 ± 1.10 at preoperative to 1.96 ± 1.06 at postoperative, further 1.19 ± 0.834 at the final follow-up. The PKP group's postoperative VAS score was significantly lower than that in PPSF + KP group (p < 0.01), but there was no significant difference in VAS score at the final follow-up (p > 0.05). As for the ODI score, the same trend was observed.

**Fig. 2:**
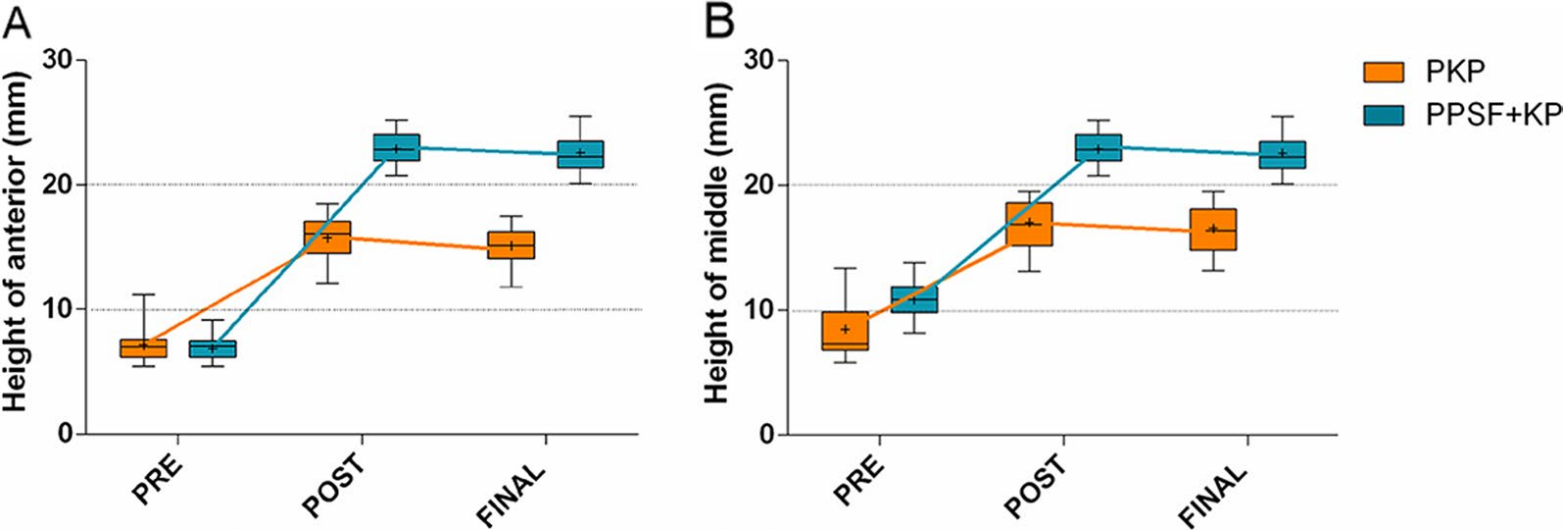
Box plots show the summary of baseline, follow-up, and changes by group. The horizontal lines in the boxplots from bottom to top show the 25th, 50th (median), and 75th percentiles. The dot in the boxplot indicates the mean. The whiskers indicate the highest and lowest values no further than 1.5 times the interquartile range. **A** Changes in the height of anterior. **B** Changes in the height of middle

The anterior and middle height vertebral bodies increased significantly after surgery. The difference in the posterior, however, was not statistically significant (Figs. 2). The HA improvement ratio (34.9 ± 9.0% vs. 63.7 ± 7.6%), the HM improvement ratio (28.4 ± 11.2% vs. 49.6 ± 7.7%), the Cobb’s angle improvement (13.4 ± 4.3° vs. 21.4 ± 5.3°) and the VWR improvement ratio (30.4 ± 11.5% vs. 52.8 ± 12.7%) were all higher in PPSF + KP group than that in PKP group (p < 0.01). The PPSF + KP group recovered faster than the PKP group (p < 0.01). **(**Table 3).

**Table 3:** Comparison of clinical results and radiographic data between the two groups at the final follow-up ($\bar{\text{x}} \pm {\text{s}}$)

Variable	PKP group	PPSF + KP group	P value
Vertebral body height ratios improvement (%)^a^			
Anterior	34.9 ± 8.9	63.6 ± 7.6	< 0.01
Middle	28.4 ± 11.2	49.6 ± 7.7	< 0.01
Posterior	5.6 ± 4.5	3.3 ± 2.7	0.382
Cobb’s angle improvement	11.9 ± 3.9	18.7 ± 4.5	< 0.01
VWR improvement (%)^b^	35.4 ± 10.7	64.6 ± 4.9	< 0.01
Cement leakage (N)	4	3	0.818
Adjacent vertebral fractures (N)	2	1	0.961

Values are expressed in mean ± standard deviation

*N* number

^a^Vertebral body height ratio (%) = (fractured vertebral body height/normal vertebral body height) × 100%

^b^VWR, Vertebral wedge ratio (%) = (fractured vertebral body anterior height/fractured vertebral body posterior height) × 100%

## Complications

Asymptomatic cement leakage occurred in 4 cases in PKP group and 3 cases in PPSF + KP group, whereas adjacent vertebral fractures occurred in 2 cases in PKP group and 1 case in PPSF + KP group. There was no statistically striking difference in number of asymptomatic cement leakage and adjacent vertebral fractures between our two groups. At the same time, no pedicle screw implantation failures were found during the follow-up process. There were no other severe issues.

### Illustrative cases

**Fig. 3:**
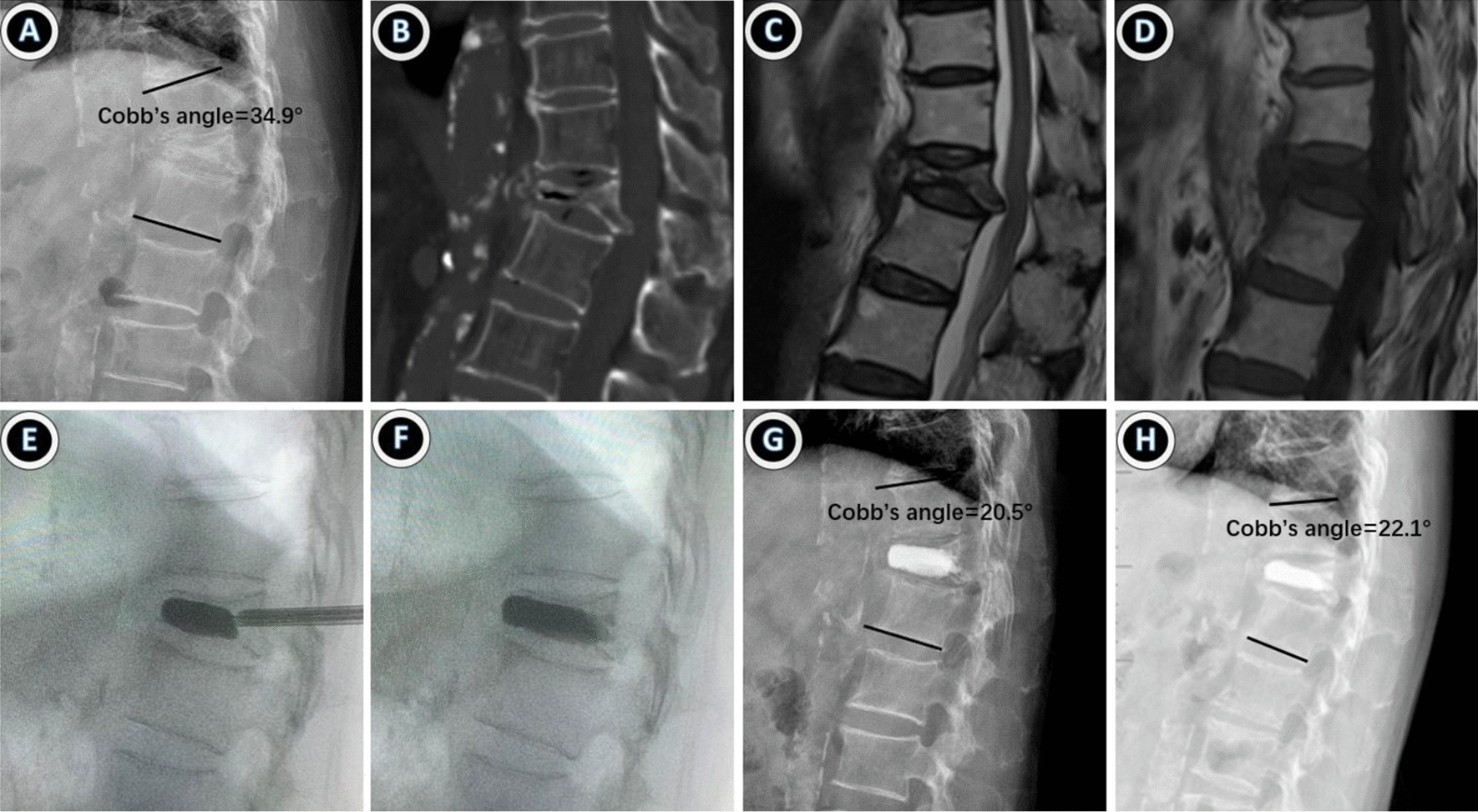
An 84-year-old woman who had T12 sOVCF with nonunion was treated with PKP. **A**–**D** The preoperative X-ray, CT and MRI showed a severe osteoporotic vertebral compression fracture with nonunion. **E**, **F** The compressed vertebral body was completely dilated during surgery using balloons and then bone cement was injected. **G**, **H** The postoperative and final follow-up X-ray displayed the vertebral height and the Cobb’s angle was well recovered

**Fig. 4:**
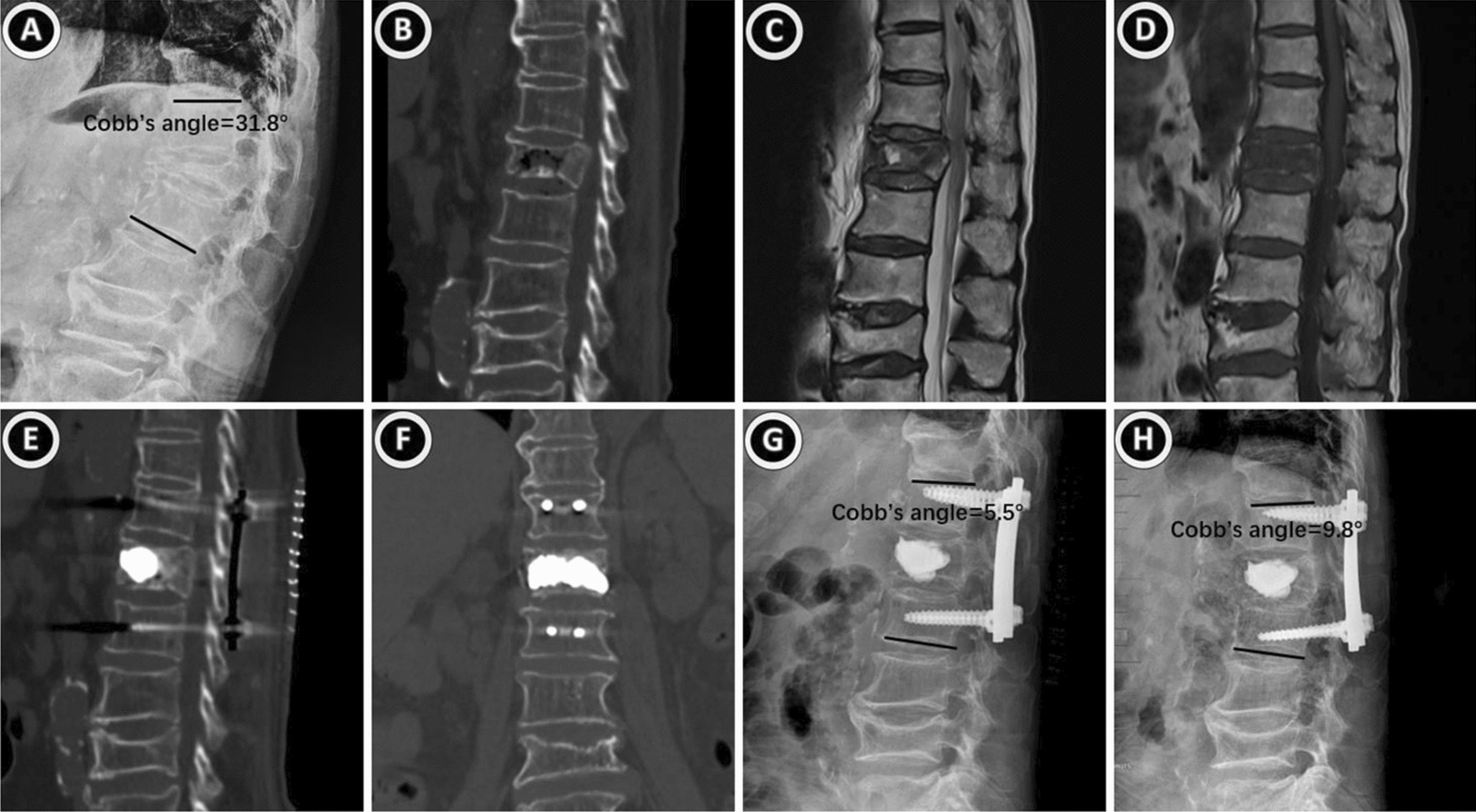
An 89-year-old woman who had L1 sOVCF with nonunion was treated with PPSF + KP. **A**–**D** The preoperative X-ray, CT and MRI showed a severe osteoporotic vertebral compression fracture with nonunion. **E**, **F** Her postoperative CT scan revealed that bone cement was evenly distributed throughout the vertebral body. **G**, **H** The postoperative and final follow-up X-ray displayed the vertebral height and the Cobb’s angle was well recovered

Illustrative cases of PKP and PPSF + KP group are shown in Figs. 3 and 4, including preoperative, intraoperative and postoperative imaging data. After PPSF + KP surgery, vertebral body height and local kyphotic angle recovered greatly, although PKP can also restore vertebral height but not as well as PPSF + KP.

## Discussion

Many patients with osteoporotic vertebral compression fractures are initially treated conservatively with bed rest and analgesia [4]. However, patients who suffered sOVCF with nonunion, have persistent back pain, progressive vertebral collapse, kyphosis and fracture activity even after conservative treatment [15, 16]. The sOVCF with nonunion that does not heal over time is a chronic source of pain and disability [17]. In the treatment of sOVCF with nonunion, open reduction and transpedicular screw internal fixation combined with PKP surgery can better restore vertebral height and anatomical sequence. However, the trauma of open reduction and transpedicular screw internal fixation is big, and patients may have obvious postoperative pain [18]. Furthermore, those patients are older and have osteoporosis, which can lead to internal fixation loosening [19, 20]. PKP has been reported to treat ordinary OVCF with nonunion and get a satisfactory outcome [21]. Nevertheless, the treatment of severe vertebral compression fractures with loss of more than two-thirds of normal vertebral height remains unclear now [10]. Currently, the commonly used surgical treatment for these patients is PKP, which can provide early pain relief, avoid long-term bed rest for the patient, and provide a satisfactory outcome with little surgical trauma and less bleeding. However, it is difficult to treat elderly patients who are suffering severe OVCF. Some doctors have also applied PKP surgery to the treatment of patients with severe OVCF. So, our aim is to evaluate the safety and efficacy of PKP and PPSF + KP in treating sOVCF with nonunion.

In this study, both PKP and PPSF + KP achieved good clinical outcomes and significantly improved quality of life. After surgery, the VAS score and ODI index were significantly lower, indicating that the two surgical methods were effective. However, on the first postoperative day, the VAS score and ODI index of PKP group were significantly lower than those of PPSF + KP group, possibly due to PPSF + KP causing damage to the back muscles and soft tissues during the operation. However, there was no significant difference at the final follow-up, which could be attributed to muscle and soft tissue injury recovery at the end of the follow-up. Furthermore, PKP group's operation time, blood loss, hospital stays, and operation costs were significantly lower than those in PPSF + KP group, demonstrating the superiority of PKP. Long-term bed rest in osteoporosis patients may exacerbate bone loss. In comparison to PPSF + KP, PKP allows patients to get out of bed earlier and break the vicious cycle. Based on the satisfactory outcome of both two groups, PKP has more advantages in controlling surgical trauma and operation cost compared to PPSF + KP.

Several studies have demonstrated that both PKP and PPSF + KP can effectively correct kyphosis and improve vertebral height [22, 23]. The height of the compressed vertebral body was effectively recovered in both groups during this study, which was consistent with the results reported in the literature [22]. The HA and HM recovery rates, cobb's angle and VWR in PPSF + KP was improved better than that in PKP group. The HA of the injured vertebrae in both groups recovered significantly after surgery compared to before surgery, and was lost to some extent during follow-up, but PPSF + KP group recovered better than PKP group. These findings suggest that PPSF + KP is more effective in restoring vertebral height and correcting kyphosis. In our study, we did not remove pedicle screws considering the older age of the patients and their good recovery, and no failure of pedicle screw implantation was observed during follow-up.

Bon cement leakage is one of the most common and potentially fatal complications of PKP surgery. The high degree of compression of the fractured vertebral body and the presence of structural defects such as fissures increase the risk of intraoperative bone cement leakage, particularly anterior leakage, in patients with sOVCF with nonunion [11, 24]. However, when PKP surgery is performed on patients with sOVCF with nonunion, due to the existence of structural defects, enough bone cement should be injected into the intravertebral fissure during surgery, and the bone cement must be fully occluded with the vertebral bone. A sufficient occlusion can ensure the stability of the bone cement in the vertebral body and restore the mechanical stability of the non-union injured vertebra. It can also achieve a good pain relief effect and allow early postoperative rehabilitation training to avoid serious complications such as loosening, displacement and prolapse of the bone cement [24]. After fracture reduction, there was a large cavity space in the injured vertebra in PPSF + KP group, allowing more bone cement to be injected.

Another major concern of PKP surgery is adjacent vertebral fractures. There is currently no agreement on whether PKP increases the possibility of adjacent vertebral fractures [25–27]. The incidence of adjacent vertebral fractures in this study was 7.41% in PKP and 4.17% in PPSF + KP group, with no statistically striking difference between the two groups. Both age and progressive bone mass loss are substantial risk factors for fracture. Regular anti-osteoporosis treatment should be performed before and after surgery for patients at high risk of osteoporosis to prevent adjacent vertebral fractures [28]. Furthermore, neither group experienced any other serious complications, such as spinal symptoms.

Kyphosis in sOVCF with nonunion patients is different from vertebral blowout fracture or degenerative kyphosis [6]. These patients frequently have severe osteoporosis, and kyphosis is frequently caused by vertebral body collapse and spinal instability [29]. After anesthesia, manipulation and postural reduction can often effectively correct the kyphosis. Therefore, the role of pedicle screws is not mainly to open and directly correct kyphosis, but to share the load pressure of the anterior column of the vertebral body after kyphosis correction, improve the stability of the spine in the fracture area, and reduce the stress borne by the bone cement reinforcement of the affected vertebrae, thereby preventing re-collapse of the anterior column of the vertebral body and bone cement displacement after surgery [23]. Furthermore, excessive distraction should be avoided during intraoperative procedures to reduce the risk of cutting the vertebral body and loosening the pedicle screws. At present, the required surgical treatment for patients with sOVCF combined with nonunion is unclear and needs to be decided based on a combination of factors such as the mechanism of injury, age, previous physical condition and mobility of the patient. In conjunction with this study, we give some suggestions for the choice of surgical procedure. For patients who are relatively young, with good pre-injury mobility, relatively good general condition, able to tolerate open surgery, have certain requirements for restoring the ability to live a normal life after surgery, and with severe OVCF and severe kyphosis deformity, PPSF + KP is recommended for early restoration of vertebral height and spinal column force line, stabilization of the fracture, and alleviation of pain, which is conducive to recovery of the patient's spinal function in the later stage of the disease. In patients who are older, in poorer general condition, with weaker mobility, with multiple comorbid medical conditions, or with severe osteoporosis, and who are assessed to have a higher risk of failure of pedicle screws, PKP surgery can be offered in conjunction with the patient's request, and can also achieve better outcomes.

For sOVCF with nonunion and patients with nerve canal symptoms, whether vertebral canal decompression is required depending on the degree of neurological symptoms and convex corrected after preoperative stenosis can be improved. Patients with sOVCF with nonunion is frequently the result of anterior flange height loss, collapse of the anterior column, and vertebral strength instability caused by relative stenosis [30]. The contraction of the posterior longitudinal ligament and the ligamentum flavum can be restored after restoring the height of the anterior margin of the vertebral body and correcting the kyphosis angle, and the spinal stenosis can be effectively improved.

### Limitations

The limitation of this study is the limited number of cases included due to the relatively low prevalence of sOVCF with nonunion. Future studies should involve more patients. Also, no data were collected to further evaluate the preoperative osteoporosis of the patients. Further investigations should ideally assess patients for preoperative osteoporosis, which would likely be related to spinal stability.

## Conclusion

Our study found that both PKP and PPSF + KP can achieve satisfactory efficacy in the treatment of sOVCF with nonunion. PPSF + KP outperforms PKP in terms of correcting kyphosis angle and restoring vertebral height. However, the trauma in PPSF + KP group was greater, the postoperative incision pain was noticeable. On the other hand, PKP causes less trauma to patients, allows them to get out of bed earlier, and offers more benefits in terms of operation time, intraoperative blood loss, hospital stays, and operation costs. These two surgical methods are effective and feasible for sOVCF with nonunion patients. However, A larger multicenter big sample randomized controlled trial is needed to determine which approach is more effective in the long run.

## Data Availability

The datasets used and/or analyzed during the current study are available from the corresponding author on reasonable request.
